# Pleasant Odor Decreases Mouse Anxiety-like Behaviors by Regulating Hippocampal Endocannabinoid Signaling

**DOI:** 10.3390/ijms251910699

**Published:** 2024-10-04

**Authors:** Jia-Rui Bi, Hai-Wei Zha, Qing-Lin Gao, Hui Wu, Zhen-Jiang Liu, Dong Sun

**Affiliations:** 1 National Engineering Laboratory for AIDS Vaccine, School of Life Sciences, Jilin University, Changchun 130012, China; bijr9921@mails.jlu.edu.cn (J.-R.B.);; 2 Key Laboratory for Molecular Enzymology and Engineering, The Ministry of Education, School of Life Sciences, Jilin University, Changchun 130012, China

**Keywords:** linalool, 2-phenylethanol, anxiety-like behaviors, retrograde endocannabinoid signaling

## Abstract

Anxiety disorder is one of the most common neuropsychiatric disorders, and affects many people’s daily activities. Although the pathogenesis and treatments of anxiety disorder have been studied for several decades, the underlying mechanisms remain elusive. Here, we provide evidence that olfactory stimuli with inhaled linalool or 2-phenylethanol decreased mouse anxiety-like behaviors and increased the activities of hippocampal dentate granule cells (DGCs). RNA-sequencing analysis identified retrograde endocannabinoid signaling, which is a critical pathway for mood regulation and neuron activation, is altered in the hippocampus of both linalool- and 2-phenylethanol-exposed mice. Further studies found that selective inhibition of endocannabinoid signaling by injecting rimonabant abolished the activation of DGCs and the anxiolytic effect induced by linalool or 2-phenylethanol. Together, these results uncovered a novel mechanism by which linalool or 2-phenylethanol decreases mouse anxiety-like behaviors and increases DG activity likely through activating hippocampal retrograde endocannabinoid signaling.

## 1. Introduction

Anxiety is a normal emotion responding to specific events or stimuli, which cause feelings of irritability, tenseness or restlessness. Everyone has the experience of anxiety during their daily lives. However, when anxiety cannot be controlled, occurs frequently and lasts for a long time, it will result in an anxiety disorder. People with an anxiety disorder often feel intense and excessive fear and worry, as well as experience cognitive deficits [1,2]. In animal models, mice with high levels of anxiety-like behaviors usually exhibit less exploration in illuminated, open and/or elevated areas [3]. Although the pathogenesis of anxiety disorders has been studied for many years and effective treatments for anxiety disorders have been used clinically, the underlying mechanisms remain largely unclear.

The dentate gyrus (DG), which is a “gate” that controls information flow into the hippocampus from the entorhinal cortex (EC), plays important roles in regulating mood. A dysfunctional DG contributes to the development of many neuropsychiatric disorders, including anxiety disorders and depression [4,5]. A growing body of evidence indicated that manipulations of hippocampal dentate granule cells (DGCs) or associated circuits are involved in regulating emotional responses. Direct activation of DGCs by optogenetic or chemogenetic methods suppresses mouse anxiety-like behaviors [6,7]. Indirect activation of DGCs by stimulating EC neurons, which is a critical pathway upstream of EC–DG circuitry, attenuates mouse depressive-like behaviors [8]. Additionally, animal training (e.g., physical exercise and environmental enrichment) and medications that reduce depression and anxiety also implicate DGCs [9,10,11,12].

It is reported that patients with anxiety show abnormalities in the olfactory discrimination task [13]. Inhaling scents could influence brain activities and have physiological effects on mood, social interaction and cognitive functions [14,15,16]. For example, exposure to an unpleasant odor usually induces negative mood and anxiety, whereas smelling a pleasant odor of essential oils will have an anxiolytic effect [17]. Thus, in addition to psychotherapy and medications for anti-anxiety, aromatherapy with essential oils is increasingly becoming a popular manner to relax and calm anxiety. Nevertheless, the underlying mechanisms by which olfactory stimuli regulate anxiety are not well understood.

Here, we provide evidence that linalool or 2-phenylethanol, which are two major components of essential oils, regulates mouse anxiety-like behaviors. Young adult mice exposed to linalool or 2-phenylethanol exhibit a significant decrease in anxiety-like behaviors as compared to control mice in a home cage. Immunostaining analysis in brain sections revealed that linalool or 2-phenylethanol stimulus largely increased the activities of DGCs. Additionally, we explored the mechanisms regarding how olfactory stimuli alleviate anxiety-like behaviors and promote DG activation by using RNA-sequencing analysis. Among the dysregulated genes and enriched signaling pathways, we found that retrograde endocannabinoid signaling is altered in the hippocampus of both linalool- and 2-phenylethanol-treated mice. Moreover, selective inhibition of endocannabinoid signaling by injecting rimonabant abolished the anxiolytic effect and DG activation induced by linalool and 2-phenylethanol. Thus, these results uncover an unrecognized mechanism of how a pleasant odor suppresses mouse anxiety-like behaviors, likely by activating retrograde endocannabinoid signaling in the hippocampus.

## 2. Result

### 2.1. Decreased Mouse Anxiety-like Behaviors by Inhaled Linalool or 2-Phenylethanol

**Figure 1:**
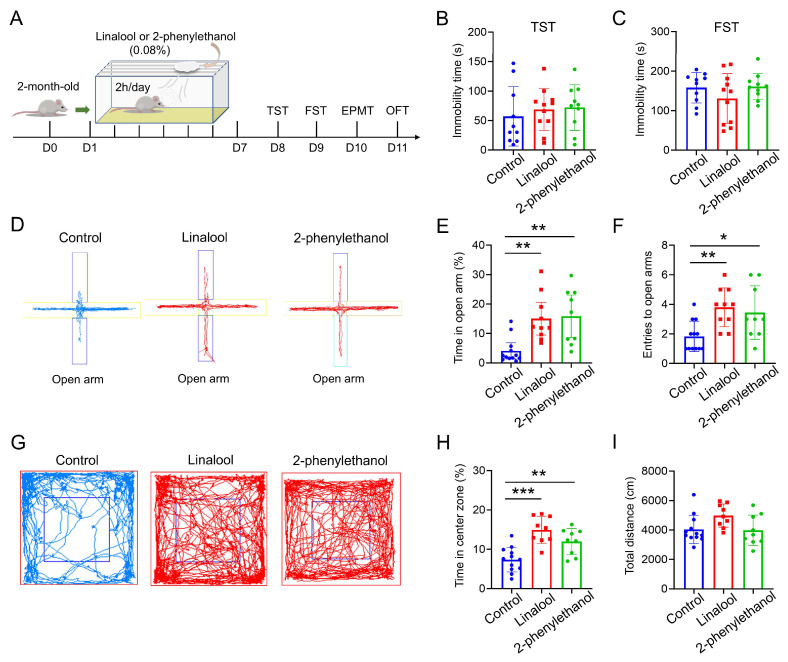
Inhaled linalool or 2-phenylethanol reduces mouse anxiety-like behaviors. (**A**) Schematic diagram of the experimental design for olfactory stimuli and subsequent behavioral tests in WT mice. (**B**,**C**) Quantifications of the immobility time of the TST (**B**) and FST (**C**) in control, and linalool- and 2-phenylethanol-exposed mice. *n* = 10 mice in the control group, *n* = 11 mice in the linalool group and *n* = 10 mice in the 2-phenylethanol group. (**D**) Representative tracing images of the EPMT in control, and linalool- and 2-phenylethanol-exposed mice. (**E**,**F**) Quantifications of the percentage of open arm duration time over total time (**E**) and the number of entries to the open arm (**F**) in the EPMT. *n* = 12 mice in the control group, *n* = 10 mice in the linalool group and *n* = 9 mice in the 2-phenylethanol group. * *p* < 0.05. ** *p* < 0.01. One-way ANOVA followed by Tukey’s multiple comparisons test. (**G**) Representative tracing images of the OFT in control, and linalool- and 2-phenylethanol-treated mice. (**H**,**I**) Quantifications of the percentage of center duration time over total time (**H**) and total distance (**I**) in the OFT. *n* = 12 mice in the control group, *n* = 9 mice in the linalool group and *n* = 9 mice in the 2-phenylethanol group. ** *p* < 0.01. *** *p* < 0.001. One-way ANOVA followed by Tukey’s multiple comparisons test. Data in (**B**,**C**,**E**,**F**,**H**,**I**) are presented as the mean ± SD.

To investigate the effects of a pleasant odor on emotional responses, 2-month-old mice were exposed to 0.08% linalool or 2-phenylethanol (2 h/day), which are two major components in essential oils, for 7 consecutive days, and subsequently subjected to behavioral tests at indicated times (Figure 1A). As shown in Figure 1B, mice exposed to linalool or 2-phenylethanol exhibited a similar inactive time in a tail suspension test (TST) as compared to control mice in a home cage, suggesting that linalool or 2-phenylethanol stimulus had little effect on depressive-like behaviors. This view was further confirmed by a forced swim test (FST), which showed comparable immobility time among the three groups of mice (Figure 1C).

Next, we asked whether linalool or 2-phenylethanol regulates anxiety-like behaviors in mice. To this end, we first performed an elevated plus maze test (EPMT) in control, and linalool- and 2-phenylethanol-exposed mice. Interestingly, linalool or 2-phenylethanol significantly increased the open arm duration time and entries as compared with those of control mice (Figure 1D–F), suggesting a decrease in anxiety-like behavior by pleasant odor stimuli. Additionally, linalool- or 2-phenylethanol-exposed mice also displayed greater center duration time than control mice in an open field test (OFT); however, total distance was indistinguishable among the three groups of mice, excluding the possibility of alteration in locomotive activity by olfactory stimuli (Figure 1G–I). Thus, these results suggest that 7 days of linalool or 2-phenylethanol treatments decreased anxiety-like behaviors but has little effect on depressive-like behaviors in mice.

### 2.2. Increased Neuronal Activity in the DG by Linalool or 2-Phenylethanol Stimulus

**Figure 2:**
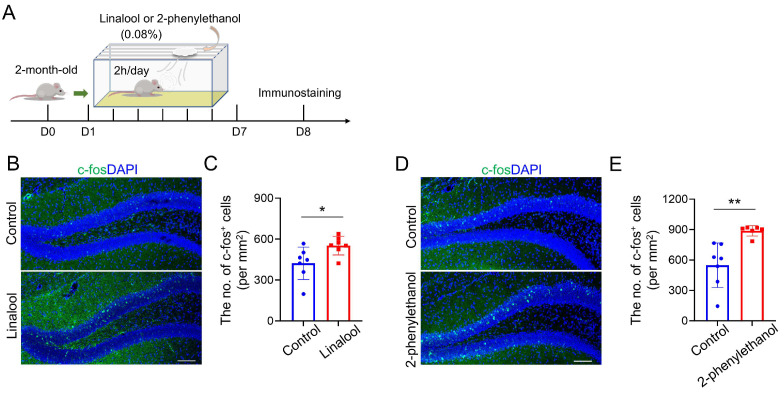
Linalool or 2-phenylethanol stimulus promotes the activation of hippocampal DGCs. (**A**) Schematic diagram of the experimental design for olfactory stimuli and subsequent immunostaining analysis in WT mice. (**B**) Immunostaining of c-fos in the DG of control and linalool-treated mice. Scale bar = 100 μm. (**C**) Quantification of the number of c-fos^+^ cells in the DG per mm^2^; *n* = 7 brain sections from four control mice and *n* = 7 brain sections from four linalool-exposed mice. * *p* < 0.05. Student’s *t*-test. (**D**) Immunostaining of c-fos in the DG of control and 2-phenylethanol-treated mice. Scale bar = 100 μm. (**E**) Quantification of the number of c-fos^+^ cells in the DG per mm^2^; *n* = 7 sections from four control mice and *n* = 6 sections from three 2-phenylethanol-exposed mice. ** *p* < 0.01. Student’s *t*-test. Data in (**C**,**E**) are presented as the mean ± SD.

Given that the DG is a critical regulator of mood [4], activating DGCs reduces mouse anxiety-like behaviors [6,7]. We, therefore, determined if olfactory stimuli changed neuronal activity in the DG. Mice were exposed to linalool or 2-phenylethanol for 7 consecutive days. One day after the last olfactory stimulation, brain samples of each group of mice were collected and subjected to immunostaining analysis using antibodies against c-fos, which is a commonly used marker for neuron activation (Figure 2A) [18]. As shown in Figure 2B,C, the number of c-fos^+^ cells was largely increased in the DG of linalool-exposed mice as compared to control mice. In line with this observation, 2-phenylethanol also increased c-fos^+^ cell density in the DG (Figure 2D,E). Thus, these results suggest that 7 days of linalool or 2-phenylethanol stimulus promotes neuron activation in the DG.

### 2.3. Altered Retrograde Endocannabinoid Signaling in the Hippocampus by Linalool and 2-Phenylethanol Stimuli

**Figure 3:**
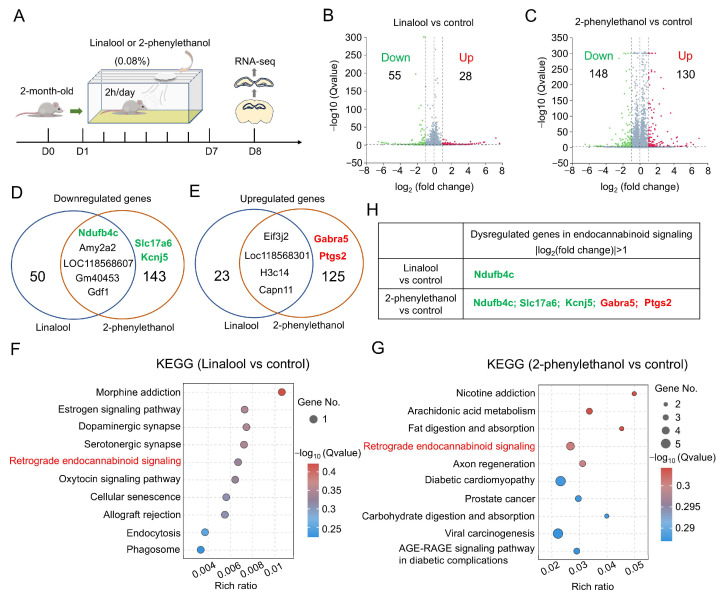
Retrograde endocannabinoid signaling is altered by linalool or 2-phenylethanol stimulus. (**A**) Schematic diagram of the experimental design for RNA-sequencing analysis in the hippocampus of linalool- or 2-phenylethanol-exposed mice. (**B**,**C**) Volcano plots of differentially expressed genes in the hippocampus of linalool-treated mice over control mice (**B**) and 2-phenylethanol-treated mice over control mice (**C**). (**D**,**E**) Venn maps of downregulated genes (**D**) and upregulated genes (**E**) between linalool- and 2-phenylethanol-treated mice. (**F**,**G**) Enriched KEGG pathways of dysregulated genes in linalool- (**F**) and 2-phenylethanol-treated (**G**) mice. (**H**) A table of dysregulated genes involved in retrograde endocannabinoid signaling for linalool- or 2-phenylethanol-exposed mice. The green color labels downregulated genes and the red color labels upregulated genes.

To understand how linalool and 2-phenylethanol suppress anxiety-like behaviors and increase DG activity, we performed RNA-sequencing analysis in the hippocampus of control, and linalool- and 2-phenylethanol-exposed mice (Figure 3A). As shown in Figure 3B,C and S1, S2, linalool induced 55 downregulated and 28 upregulated genes, and 2-phenylethanol induced 148 downregulated genes and 130 upregulated genes in the hippocampus as compared to control mice. Among these dysregulated genes, five downregulated genes and four upregulated genes overlapped in linalool- and 2-phenylethanol-exposed mice (Figure 3D,E), suggesting that linalool and 2-phenylethanol’s functions in anti-anxiety and DG activation may share the same mechanism. To test this view, we further analyzed the enriched KEGG pathways with dysregulated genes in each group of mice. Interestingly, we identified retrograde endocannabinoid signaling, which is a key regulator of mood and neuronal activity [19,20], is altered by linalool and 2-phenylethanol stimuli (Figure 3F,G). Notably, the downregulated genes (*Ndufb4c*, *Slc17a6* and *Kcnj5*) and upregulated genes (*Gabra5* and *Ptgs2*) are responsible for retrograde endocannabinoid signaling (Figure 3H). Thus, these results led to the hypothesis that linalool and 2-phenylethanol regulate anxiety-like behaviors and DG activity likely through retrograde endocannabinoid signaling.

### 2.4. DG Activation and Anxiolytic Effect in Linalool- or 2-Phenylethanol-Exposed Mice Abolished by Inhibiting Retrograde Endocannabinoid Signaling

**Figure 4:**
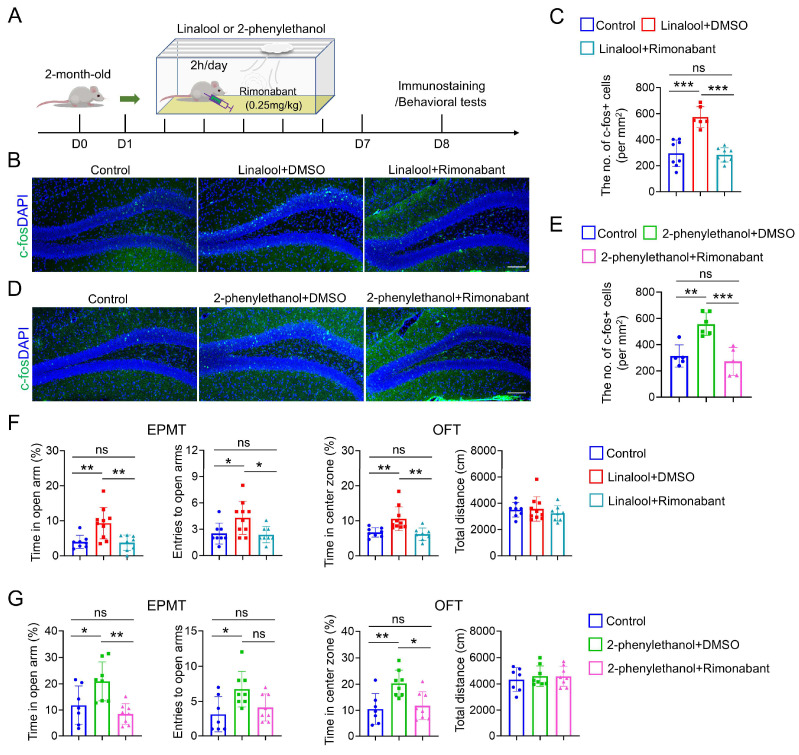
Retrograde endocannabinoid signaling is required for DG activation and the anxiolytic effect by pleasant odor stimuli. (**A**) Schematic diagram of the experimental design for olfactory stimuli, rimonabant injection and subsequent immunostaining analysis and behavioral tests in WT mice. (**B**) Immunostaining of c-fos in the DG of control, linalool + DMSO and linalool + rimonabant mice. Scale bar = 100 μm. (**C**) Quantification of the number of c-fos^+^ cells in DG per mm^2^; *n* = 8 sections from four control mice, *n* = 6 sections from three linalool + DMSO mice and *n* = 8 sections from four linalool + rimonabant mice. *** *p* < 0.001, ns = no significant difference. One-way ANOVA followed by Tukey’s multiple comparisons test. (**D**) Immunostaining of c-fos in the DG of control, 2-phenylethanol + DMSO and 2-phenylethanol+ rimonabant mice. Scale bar = 100 μm. (**E**) Quantification of the number of c-fos^+^ cells in the DG per mm^2^; *n* = 5 sections from three control mice, *n* = 6 sections from three 2-phenylethanol + DMSO mice and *n* = 5 sections from three 2-phenylethanol + rimonabant mice. ** *p* < 0.01, *** *p* < 0.001, ns = no significant difference. One-way ANOVA followed by Tukey’s multiple comparisons test. (**F**) Evaluation of anxiety-like behaviors by the EPMT and OFT in control, linalool + DMSO and linalool + rimonabant mice. *n* = 8 mice for the control group, *n* = 10 mice for the linalool + DMSO group and *n* = 8 mice for the linalool+ rimonabant group. * *p* < 0.05, ** *p* < 0.01, ns = no significant difference. One-way ANOVA followed by Tukey’s multiple comparisons test. (**G**) Evaluation of anxiety-like behaviors by the EPMT and OFT in control, 2-phenylethanol + DMSO and 2-phenylethanol + rimonabant mice. *n* = 7 mice for the control group, *n* = 8 mice for the 2-phenylethanol + DMSO group and *n* = 8 mice for the 2-phenylethanol + rimonabant group. * *p* < 0.05, ** *p* < 0.01, ns = no significant difference. One-way ANOVA followed by Tukey’s multiple comparisons test. Data in (**C**,**E**,**F**,**G**) are presented as the mean ± SD.

Given that activating endocannabinoid signaling normally produces anxiolytic effects [21], we next asked whether retrograde endocannabinoid signaling is required for DG activation and anti-anxiety effects by linalool or 2-phenylethanol stimulus. To address this question, rimonabant, which is a selective antagonist of cannabinoid type 1 receptor [22], was applied in linalool- or 2-phenylethanol-exposed mice. As shown in Figure 4A, rimonabant was injected into mice before daily linalool or 2-phenylethanol exposure, and each group of mice were subjected to immunostaining analysis and behavioral tests at the indicated time. Interestingly, rimonabant treatment induced a significant decrease in c-fos^+^ DG neurons in linalool-exposed mice as compared to linalool + DMSO mice (Figure 4B,C). In addition, the number of c-fos^+^ DGCs was also largely decreased in 2-phenylethanol + rimonabant mice, which restored numbers to a comparable level with that in control mice (Figure 4D,E).

Next, we examined anxiety-like behaviors in control, linalool + DMSO and linalool + rimonabant mice. As shown in Figure 4F, compared to control mice, open arm duration time and entries in the EPMT and center time in the OFT were all increased in linalool + DMSO mice but not in linalool + rimonabant mice, suggesting a necessary role of retrograde endocannabinoid signaling in linalool-induced anti-anxiety. Furthermore, similar phenotypes were also detected in 2-phenylethanol + rimonabant mice as compared to control mice (Figure 4G), providing additional support for the view that the anxiolytic effect of 2-phenylethanol requires retrograde endocannabinoid signaling.

## 3. Discussion

Olfactory stimuli are implicated in many aspects of brain functions and diseases [23,24,25,26]. Here, we provide evidence that inhaled linalool or 2-phenylethanol plays an important role in regulating mouse anxiety-like behaviors. Chronic exposure to linalool or 2-phenylethanol largely activated DGCs and decreased innate/basal anxiety-like behaviors in mice. Notice that inhaled 2-phenylethanol could decrease anxiety-like behaviors in stressed mice upon chronic corticosterone treatment [27], and oral administration of an essential oil containing linalool also had anxiolytic effects in restraint-stressed mice [28]. Thus, in combination with other literature reports, our results support the view that 2-phenylethanol and linalool not only regulate stress-induced anxiety but also affect innate/basal anxiety-like behaviors in mice.

It is reported that many psychiatric disorders, including anxiety disorders, have a close relationship with olfactory abnormalities [29,30]. Clinical studies showed that patients with an anxiety disorder exhibit deficits in olfactory discrimination without changing threshold and identification ability [13]. Conversely, olfactory dysfunction caused by chronic rhinosinusitis increased the risk of anxiety and depression symptoms [31]. In animal models, impairments in smelling increased mouse anxiety-like behaviors [32]. Additionally, hyperactivity of olfactory bulb-to-medial prefrontal cortex circuitry is also responsible for anxiety in rats [33]. However, whether the hippocampus, which is another critical brain region for emotional processing, is involved in regulating the olfactory-associated anxiety state remains largely unknown. Our findings demonstrated that decreased mouse anxiety-like behaviors by linalool or 2-phenylethanol implicate the hippocampus, which was supported by the following reasons: First, inhaled linalool or 2-phenylethanol largely increased the activities of DGCs (Figure 2), which have been previously proven for their anxiolytic effect in mice [6,7]. Second, we found that both linalool and 2-phenylethanol stimuli changed transcriptional characteristics in the hippocampus (Figure 3B,C); dysregulated genes are enriched in a series of signaling pathways by KEGG analysis (Figure 3F,G), including anxiety-associated retrograde endocannabinoid signaling [34,35].

As reported [36,37], chronic treatments with cannabis bidirectionally affect anxiety in humans; an increase or decrease in anxiety may depend on cannabinoid receptor distribution. In animal models, activating endocannabinoid signaling usually has an anti-anxiety effect, whereas disruption of endocannabinoid signaling increased anxiety-like behaviors [38,39]. Although endocannabinoid signaling and associated brain regions in regulating anxiety have been extensively studies for decades, whether the anxiolytic effects induced by a pleasant odor require endocannabinoid signaling are not clear. In our study, we report by RNA-sequencing analysis that linalool or 2-phenylethanol facilitates endocannabinoid signaling in the hippocampus (Figure 3D–H). Furthermore, selective inhibition of endocannabinoid signaling by injecting rimonabant not only restored DG activity to the control level but also blocked the anxiolytic effect of linalool or 2-phenylethanol (Figure 4). However, it remains to be determined whether the activation of endocannabinoid signaling by linalool or 2-phenylethanol stimulus was specific to the hippocampus. Additionally, it is also worth exploring what mechanisms are involved in the activation of endocannabinoid signaling by pleasant odor stimuli.

## 4. Materials and Methods

### 4.1. Animals

WT (C57BL/6J) mice were purchased from Charles River company. Both male and female mice were used in this study. All mice were group-housed (maximum 5 mice per cage), and maintained on a 12 h light–dark cycle with ad libitum access to water and food. Laboratory Animal Guideline for Ethical Review of Animal Welfare (GB/T 35892-2018) was the guidance for our animal care protocols. All the experiments with animals were performed with the approval of the Institutional Animal Care and Use Committee of Jilin University (YNPZXM202201).

### 4.2. Reagents and Drug Treatment

Linalool (L812404) and 2-phenylethanol (P815996) were purchased from MACKLIN company (Shanghai, China). Both linalool and 2-phenylethanol were diluted into 0.08% with water (*v*/*v*). For odor stimuli, 2 mL of linalool or 2-phenylethanol were dropped onto cotton, allowing the mouse to smell it for 2 h per day. Control mice were littermates in a home cage without odor stimuli. Rimonabant (R125003) was purchased from Aladdin company (Shanghai, China), and 0.25 mg/kg body weight of rimonabant was used to treat (intraperitoneal injection) mice. For immunostaining, the primary antibody of c-fos (Santa Cruz, sc-271243, Dallas, TX, USA) was used for labeling neuron activation. 4′,6-diamidino-2-phenylindole (DAPI) was used for staining nuclei (1:1000, Roche, Graz Steiermark, Austria).

### 4.3. Behavioral Tests

All behavioral tests were performed as previously described with slight modifications [6,40]. Before each test, all mice were transferred to the behavioral room 2 h in advance to acclimate to the environment. All behavioral instruments were cleaned with 75% ethanol prior to each trial.

For the tail suspension test (TST), each mouse was hung by the tail using tape, where one end of the tape was secured to a horizontal bar 40 cm from the ground, thus ensuring that the animal could not climb on other objects during the assay. The last 4 min of a 6 min test were analyzed and the immobility time was calculated.

For the forced swim test (FST), each mouse was placed in a clear glass cylinder (height 30 cm, diameter 10 cm), which was filled with water (height: 20 cm, temperature: 22–25 °C). The last 4 min of a 6 min test were analyzed and the immobility time was calculated.

For the elevated plus maze test (EPMT), the EPM was placed 50 cm above the ground. Each mouse was initially placed in the center square facing one of the open arms (L × W = 30 × 5 cm). Mice movement was recorded for 5 min using an overhead camera and tracking software (Smart 3.0, Panlab Harvard Apparatus, Whitehall, PA, USA). The percentage of time spent in the open arms and the number of open arm entries were quantified.

For the open field test (OFT), each mouse was placed in a chamber (L × W × H = 50 × 50 × 50 cm) and movement was monitored for 10 min using an overhead camera. The light intensity was about 200 lux. The video was analyzed by tracking software (Smart 3.0, Panlab Harvard Apparatus). Total distance and the percentage of time spent in the center (20 × 20 cm) were quantified.

### 4.4. Immunohistochemistry and Image Analysis

Immunostaining was performed as described previously [41]. Each group of mice was anesthetized with isoflurane and perfused with 50 mL PBS followed by 50 mL 4% paraformaldehyde (PFA). Brains were post-fixed in 2% PFA overnight at 4 °C and cut into 40 μm slices using a vibratome (Leica VT1000 S, Wetzlar, Germany). For immunostaining, brain sections were washed 3 times with PBS (5 min/time) and treated with blocking buffer (10% Donkey Serum + 0.5% Triton × 100) for 1 h at room temperature, and then incubated with primary antibody (c-fos, 1:500 in PBS) overnight at 4 °C. Sections were washed 4 times with PBST and incubated with corresponding conjugated secondary antibody for 2 h at room temperature. Finally, the sections were washed 4 times with PBST; nuclei were stained with DAPI for 5 min.

### 4.5. RNA-Sequencing Analysis

RNA-sequencing analysis was performed as described previously with a slight modification [41]. After 7 days of olfactory stimuli, we extracted total RNA from the hippocampi of each group of mice at day 8 by using an MGIEasy Total RNA Extraction Kit (Cat. No. 940-000877-00, MGI, Shen Zhen, China). In this study, an agilent 2100 Bio analyzer (Agilent RNA 6000 Nano Kit, Santa Clara, CA, USA) was used to perform RNA sample QC: RNA concentration, RIN value, 28S/18S and fragment length distribution. Subsequently, RNA-seq was performed by using a BGI DNBseq platform.

### 4.6. Statistical Analysis

All results presented in this study were from at least three independent experiments. GraphPad Prism 9.0 software was used for statistical analyses. For comparisons of two groups of samples, a student’s *t*-test was used to evaluate statistical significance. For multiple comparisons of three or more groups of samples, ANOVA was used. All data are expressed as the mean ± SD and described in the figure legends. Statistical significance was defined as *p* < 0.05.

## 5. Conclusions

In summary, our results indicate that linalool or 2-phenylethanol increases DG activity and suppresses mouse anxiety-like behaviors likely through activating endocannabinoid signaling in the hippocampus.

## Data Availability

The data that support the findings of this study are available from the corresponding author upon reasonable request.

## Supplementary Materials

The following supporting information can be downloaded at: https://www.mdpi.com/article/10.3390/ijms251910699/s1.
